# Supplementary material for: Multi‐dataset identification of innovative feature genes and molecular mechanisms in keratoconus
Source: J Cell Mol Med. 2024 Sep 19;28(18):e70079. doi: 10.1111/jcmm.70079 (PMC11412914; doi:10.1111/jcmm.70079)
Supplement: Supplementary file 2 — Material S2. [file JCMM-28-e70079-s002.zip › 7.GSEA analysis/5.RNF217-AS1/KEGG_HUNTINGTONS_DISEASE.html]

Details for gene set KEGG\_HUNTINGTONS\_DISEASE[GSEA]

|  || Dataset | RNF217-AS1.group.cls#h\_versus\_l |
| Phenotype | group.cls#h\_versus\_l |
| Upregulated in class | l |
| GeneSet | KEGG\_HUNTINGTONS\_DISEASE |
| Enrichment Score (ES) | -0.24110277 |
| Normalized Enrichment Score (NES) | -1.3759539 |
| Nominal p-value | 0.11023622 |
| FDR q-value | 0.3560381 |
| FWER p-Value | 0.891 |
Table: GSEA Results Summary

  

Fig 1: Enrichment plot: KEGG\_HUNTINGTONS\_DISEASE      
 Profile of the Running ES Score & Positions of GeneSet Members on the Rank Ordered List

  

| SYMBOL | TITLE | RANK IN GENE LIST | RANK METRIC SCORE | RUNNING ES | CORE ENRICHMENT || 1 | SLC25A31 | na | 1638 | 0.379 | -0.0129 | No |
| 2 | CLTC | na | 2152 | 0.337 | 0.0029 | No |
| 3 | GRM5 | na | 3284 | 0.279 | -0.0044 | No |
| 4 | TBPL2 | na | 4999 | 0.217 | -0.0342 | No |
| 5 | PPARG | na | 5598 | 0.196 | -0.0337 | No |
| 6 | COX7B2 | na | 6236 | 0.176 | -0.0361 | No |
| 7 | GNAQ | na | 7033 | 0.152 | -0.0453 | No |
| 8 | PLCB3 | na | 7444 | 0.141 | -0.0443 | No |
| 9 | DNAI1 | na | 9814 | 0.089 | -0.1047 | No |
| 10 | CASP3 | na | 9912 | 0.088 | -0.0995 | No |
| 11 | UCP1 | na | 10055 | 0.086 | -0.0959 | No |
| 12 | HAP1 | na | 10075 | 0.085 | -0.0887 | No |
| 13 | POLR2H | na | 10375 | 0.080 | -0.0900 | No |
| 14 | COX4I2 | na | 11142 | 0.068 | -0.1060 | No |
| 15 | COX7A2L | na | 11823 | 0.058 | -0.1204 | No |
| 16 | CREB3 | na | 11909 | 0.057 | -0.1177 | No |
| 17 | RCOR1 | na | 12156 | 0.053 | -0.1200 | No |
| 18 | COX6B2 | na | 12168 | 0.053 | -0.1155 | No |
| 19 | VDAC2 | na | 12701 | 0.046 | -0.1267 | No |
| 20 | HTT | na | 13719 | 0.034 | -0.1530 | No |
| 21 | DNAI2 | na | 13846 | 0.032 | -0.1537 | No |
| 22 | SOD2 | na | 13856 | 0.032 | -0.1511 | No |
| 23 | NDUFA10 | na | 14013 | 0.031 | -0.1528 | No |
| 24 | NDUFB5 | na | 14086 | 0.030 | -0.1522 | No |
| 25 | SP1 | na | 14246 | 0.028 | -0.1542 | No |
| 26 | APAF1 | na | 14427 | 0.026 | -0.1571 | No |
| 27 | CREBBP | na | 14557 | 0.024 | -0.1587 | No |
| 28 | POLR2J3 | na | 14815 | 0.021 | -0.1642 | No |
| 29 | REST | na | 14836 | 0.021 | -0.1629 | No |
| 30 | POLR2J | na | 14839 | 0.021 | -0.1610 | No |
| 31 | COX7B | na | 14914 | 0.020 | -0.1614 | No |
| 32 | CLTA | na | 15454 | 0.014 | -0.1756 | No |
| 33 | COX6C | na | 15464 | 0.014 | -0.1746 | No |
| 34 | NDUFV1 | na | 15508 | 0.014 | -0.1746 | No |
| 35 | NDUFS6 | na | 15562 | 0.013 | -0.1749 | No |
| 36 | NDUFA1 | na | 15926 | 0.010 | -0.1845 | No |
| 37 | POLR2C | na | 16068 | 0.008 | -0.1879 | No |
| 38 | NDUFS4 | na | 16079 | 0.008 | -0.1874 | No |
| 39 | NDUFS1 | na | 16420 | 0.004 | -0.1969 | No |
| 40 | CREB1 | na | 16423 | 0.004 | -0.1965 | No |
| 41 | NDUFB1 | na | 16574 | 0.003 | -0.2006 | No |
| 42 | CREB3L3 | na | 16617 | 0.003 | -0.2016 | No |
| 43 | COX6B1 | na | 16711 | 0.002 | -0.2041 | No |
| 44 | SIN3A | na | 16865 | -0.000 | -0.2085 | No |
| 45 | COX7C | na | 16867 | -0.000 | -0.2086 | No |
| 46 | POLR2A | na | 16956 | -0.001 | -0.2110 | No |
| 47 | NDUFB3 | na | 16984 | -0.001 | -0.2116 | No |
| 48 | COX5B | na | 17009 | -0.002 | -0.2122 | No |
| 49 | SDHD | na | 17106 | -0.003 | -0.2147 | No |
| 50 | UQCRC2 | na | 17167 | -0.003 | -0.2162 | No |
| 51 | UQCR11 | na | 17489 | -0.006 | -0.2249 | No |
| 52 | NDUFAB1 | na | 17544 | -0.007 | -0.2258 | No |
| 53 | NDUFC1 | na | 17617 | -0.007 | -0.2272 | No |
| 54 | NDUFS5 | na | 17643 | -0.008 | -0.2273 | No |
| 55 | COX7A2 | na | 17707 | -0.008 | -0.2284 | No |
| 56 | SDHA | na | 17733 | -0.008 | -0.2283 | No |
| 57 | NDUFA6 | na | 17794 | -0.009 | -0.2292 | No |
| 58 | NDUFB4 | na | 17944 | -0.010 | -0.2326 | No |
| 59 | NDUFS8 | na | 17948 | -0.010 | -0.2318 | No |
| 60 | COX6A1 | na | 18053 | -0.011 | -0.2338 | No |
| 61 | UQCRC1 | na | 18155 | -0.012 | -0.2356 | No |
| 62 | POLR2I | na | 18217 | -0.013 | -0.2362 | No |
| 63 | DNAL4 | na | 18232 | -0.013 | -0.2354 | No |
| 64 | HDAC1 | na | 18248 | -0.013 | -0.2346 | No |
| 65 | POLR2J2 | na | 18415 | -0.015 | -0.2381 | No |
| 66 | NDUFB9 | na | 18519 | -0.016 | -0.2397 | Yes |
| 67 | UQCRQ | na | 18543 | -0.016 | -0.2389 | Yes |
| 68 | CASP8 | na | 18593 | -0.016 | -0.2388 | Yes |
| 69 | COX8A | na | 18621 | -0.017 | -0.2381 | Yes |
| 70 | NDUFS3 | na | 18677 | -0.017 | -0.2381 | Yes |
| 71 | NDUFV3 | na | 18696 | -0.017 | -0.2371 | Yes |
| 72 | NDUFV2 | na | 18702 | -0.017 | -0.2356 | Yes |
| 73 | NDUFB8 | na | 18716 | -0.018 | -0.2344 | Yes |
| 74 | COX6A2 | na | 18729 | -0.018 | -0.2331 | Yes |
| 75 | SLC25A5 | na | 18805 | -0.018 | -0.2336 | Yes |
| 76 | NDUFA4 | na | 18841 | -0.019 | -0.2329 | Yes |
| 77 | AP2B1 | na | 18948 | -0.020 | -0.2342 | Yes |
| 78 | AP2S1 | na | 19056 | -0.020 | -0.2355 | Yes |
| 79 | POLR2L | na | 19097 | -0.021 | -0.2347 | Yes |
| 80 | COX5A | na | 19181 | -0.022 | -0.2351 | Yes |
| 81 | NDUFB7 | na | 19315 | -0.023 | -0.2369 | Yes |
| 82 | DCTN2 | na | 19382 | -0.024 | -0.2366 | Yes |
| 83 | SLC25A6 | na | 19447 | -0.025 | -0.2362 | Yes |
| 84 | NDUFA9 | na | 19508 | -0.025 | -0.2356 | Yes |
| 85 | NDUFS7 | na | 19536 | -0.026 | -0.2341 | Yes |
| 86 | SLC25A4 | na | 19627 | -0.026 | -0.2343 | Yes |
| 87 | NDUFB10 | na | 19641 | -0.027 | -0.2322 | Yes |
| 88 | NDUFA7 | na | 19695 | -0.027 | -0.2313 | Yes |
| 89 | POLR2F | na | 19959 | -0.030 | -0.2362 | Yes |
| 90 | AP2M1 | na | 19983 | -0.030 | -0.2342 | Yes |
| 91 | PPID | na | 20070 | -0.031 | -0.2338 | Yes |
| 92 | CREB3L2 | na | 20073 | -0.031 | -0.2311 | Yes |
| 93 | NDUFA3 | na | 20174 | -0.032 | -0.2311 | Yes |
| 94 | POLR2G | na | 20302 | -0.033 | -0.2318 | Yes |
| 95 | UQCRH | na | 20365 | -0.033 | -0.2305 | Yes |
| 96 | TBPL1 | na | 20414 | -0.034 | -0.2288 | Yes |
| 97 | COX4I1 | na | 20433 | -0.034 | -0.2262 | Yes |
| 98 | SOD1 | na | 20467 | -0.035 | -0.2240 | Yes |
| 99 | NDUFA4L2 | na | 20479 | -0.035 | -0.2212 | Yes |
| 100 | VDAC3 | na | 20555 | -0.035 | -0.2202 | Yes |
| 101 | NDUFA8 | na | 20605 | -0.036 | -0.2183 | Yes |
| 102 | CLTB | na | 20606 | -0.036 | -0.2150 | Yes |
| 103 | NDUFB2 | na | 20644 | -0.036 | -0.2128 | Yes |
| 104 | CREB3L1 | na | 20661 | -0.036 | -0.2099 | Yes |
| 105 | COX7A1 | na | 20777 | -0.038 | -0.2098 | Yes |
| 106 | SDHC | na | 20833 | -0.038 | -0.2080 | Yes |
| 107 | HDAC2 | na | 20836 | -0.038 | -0.2045 | Yes |
| 108 | CASP9 | na | 20912 | -0.039 | -0.2032 | Yes |
| 109 | GPX1 | na | 20965 | -0.040 | -0.2011 | Yes |
| 110 | UQCRFS1 | na | 21182 | -0.042 | -0.2035 | Yes |
| 111 | DCTN4 | na | 21204 | -0.042 | -0.2003 | Yes |
| 112 | NDUFA2 | na | 21309 | -0.043 | -0.1994 | Yes |
| 113 | UQCRB | na | 21369 | -0.044 | -0.1971 | Yes |
| 114 | PLCB4 | na | 21374 | -0.044 | -0.1932 | Yes |
| 115 | DNAL1 | na | 21499 | -0.045 | -0.1927 | Yes |
| 116 | BBC3 | na | 21579 | -0.046 | -0.1908 | Yes |
| 117 | VDAC1 | na | 21769 | -0.048 | -0.1919 | Yes |
| 118 | SDHB | na | 21783 | -0.048 | -0.1878 | Yes |
| 119 | CREB3L4 | na | 21836 | -0.049 | -0.1849 | Yes |
| 120 | NDUFB6 | na | 21949 | -0.050 | -0.1836 | Yes |
| 121 | DCTN1 | na | 21980 | -0.051 | -0.1798 | Yes |
| 122 | UQCR10 | na | 21990 | -0.051 | -0.1755 | Yes |
| 123 | BAX | na | 22034 | -0.051 | -0.1721 | Yes |
| 124 | POLR2E | na | 22042 | -0.051 | -0.1676 | Yes |
| 125 | TBP | na | 22218 | -0.053 | -0.1679 | Yes |
| 126 | CYCS | na | 22416 | -0.055 | -0.1685 | Yes |
| 127 | NDUFA5 | na | 22469 | -0.056 | -0.1650 | Yes |
| 128 | AP2A1 | na | 23310 | -0.066 | -0.1833 | Yes |
| 129 | POLR2D | na | 23397 | -0.067 | -0.1797 | Yes |
| 130 | TAF4 | na | 23472 | -0.068 | -0.1757 | Yes |
| 131 | EP300 | na | 23570 | -0.069 | -0.1722 | Yes |
| 132 | CYC1 | na | 23861 | -0.073 | -0.1739 | Yes |
| 133 | TFAM | na | 24297 | -0.080 | -0.1793 | Yes |
| 134 | GRIN1 | na | 24441 | -0.082 | -0.1760 | Yes |
| 135 | NDUFC2 | na | 24639 | -0.085 | -0.1740 | Yes |
| 136 | POLR2B | na | 24796 | -0.087 | -0.1705 | Yes |
| 137 | AP2A2 | na | 24835 | -0.088 | -0.1637 | Yes |
| 138 | ITPR1 | na | 25459 | -0.098 | -0.1728 | Yes |
| 139 | NDUFS2 | na | 25676 | -0.101 | -0.1698 | Yes |
| 140 | POLR2K | na | 25798 | -0.103 | -0.1639 | Yes |
| 141 | HIP1 | na | 26025 | -0.107 | -0.1607 | Yes |
| 142 | CLTCL1 | na | 26347 | -0.113 | -0.1597 | Yes |
| 143 | GRIN2B | na | 26448 | -0.115 | -0.1521 | Yes |
| 144 | DLG4 | na | 26795 | -0.122 | -0.1510 | Yes |
| 145 | DNAH1 | na | 27011 | -0.126 | -0.1458 | Yes |
| 146 | NRF1 | na | 27124 | -0.128 | -0.1373 | Yes |
| 147 | TAF4B | na | 28585 | -0.165 | -0.1646 | Yes |
| 148 | TGM2 | na | 28791 | -0.171 | -0.1550 | Yes |
| 149 | PPARGC1A | na | 29046 | -0.177 | -0.1462 | Yes |
| 150 | DNAH2 | na | 29091 | -0.179 | -0.1312 | Yes |
| 151 | PLCB2 | na | 31149 | -0.248 | -0.1682 | Yes |
| 152 | BDNF | na | 31852 | -0.282 | -0.1628 | Yes |
| 153 | TP53 | na | 32227 | -0.304 | -0.1459 | Yes |
| 154 | PLCB1 | na | 32371 | -0.315 | -0.1214 | Yes |
| 155 | CREB5 | na | 32518 | -0.325 | -0.0960 | Yes |
| 156 | DNALI1 | na | 32987 | -0.359 | -0.0770 | Yes |
| 157 | IFT57 | na | 34235 | -0.537 | -0.0642 | Yes |
| 158 | COX8C | na | 34671 | -0.862 | 0.0016 | Yes |
Table: GSEA details [plain text format]

  

Fig 2: KEGG\_HUNTINGTONS\_DISEASE      
 Blue-Pink O' Gram in the Space of the Analyzed GeneSet

  

Fig 3: KEGG\_HUNTINGTONS\_DISEASE: Random ES distribution      
 Gene set null distribution of ES for **KEGG\_HUNTINGTONS\_DISEASE**
